# The epidemiological characteristics of liver disease in hospitalized children: a 10-year single-center retrospective study

**DOI:** 10.3389/fped.2024.1344714

**Published:** 2024-03-06

**Authors:** Fan Chen, Yuan Huang, Zhihua Huang, Feng Fang, Hua Zhou, Sainan Shu

**Affiliations:** Department of Pediatrics, Tongji Hospital, Tongji Medical College, Huazhong University of Science and Technology, Wuhan, China

**Keywords:** pediatrics, inpatients, epidemiology, liver disease, non-alcoholic fatty liver disease

## Abstract

**Background:**

This investigation aimed to examine the epidemiological characteristics of children with liver disease hospitalized for the first time between June 2012 and May 2022 in a tertiary hospital.

**Methods:**

The study retrospectively recruited children aged between 29 days and 18 years who had been hospitalized for liver disease. Clinical characteristics were categorized by age and etiology, and time trends were assessed using linear regression analysis.

**Results:**

A total of 4,313 children were recruited, with a median age of 0.7 (0.2–4.5) years, and 54.5% of the cases were in the 0–1 years age group. Infection was the primary cause of liver disease (30.0%), followed by undiagnosed cases (25.8%), biliary obstructive disease (15.9%), inherited metabolic liver disease (13.9%), and non-alcoholic fatty liver disease (NAFLD) (3.2%). Genetic diagnoses were established in 43.9% (478/1,088) of patients. The percentage of NAFLD demonstrated an upward trend from 1.2% in 2012 to 12.6% in 2022 (*p *= 0.006). In contrast, the percentage of cytomegalovirus hepatitis decreased from 13.3% in 2012 to 3.4% in 2022 (*p *= 0.002).

**Conclusions:**

Liver disease in infancy makes up the largest group in pediatric liver disease. Infection remains the leading cause of pediatric liver disease. Hospital admissions for NAFLD in children have increased rapidly over the past decade, while cytomegalovirus hepatitis has declined markedly.

## Introduction

Pediatric hepatology has branched out into nearby clinical fields, such as metabolic liver diseases and systemic diseases involving the liver (1, 2). Next-generation sequencing (NGS) has become a powerful diagnostic tool in recent years (3), particularly with the application of panel-based NGS and whole-exome sequencing (WES). The growing prevalence of gene sequencing in clinical settings has also altered the range of liver diseases (4, 5). A growing number of novel genetic diseases have been added to the list of pediatric liver disease etiologies. However, pediatric liver disease continues to be a major challenge around the world (6). Certain liver diseases that manifest during childhood serve as antecedents to chronic liver disease, cirrhosis, and hepatocellular carcinoma in adulthood (7). Prompt identification and intervention have the potential to significantly modify the progression of the ailment and enhance the patient's overall well-being (8). Liver disease is not mentioned in the top fifteen causes of death for children, however, approximately two million fatalities per year are attributed to liver disease, which accounts for 4% of global mortality (9). According to 2000 data, approximately 15,000 children in the United States are hospitalized annually due to liver disease (10). To the best of our knowledge, the majority of current research focuses on particular liver disorders, including cholestasis and inherited liver disease, or a particular demographic. Further elaboration is necessary to estimate disease prevalence effectively and prioritize healthcare policies.

Hubei Province is situated in central China and has a resident population of 58.44 million people, making it the tenth-largest province in China by population. To further understand the epidemiological characteristics of pediatric liver disease, to facilitate early diagnosis and treatment, a retrospective study on children admitted to the hospital with liver disease was conducted at Tongji Hospital, Tongji Medical College, Huazhong University of Science and Technology between June 2012 and May 2022. Our study aims to analyze the clinical spectrum of a large cohort of hospitalized children with liver disease and investigate the time trend. To the best of our knowledge, this is the first comprehensive elucidation of the disease spectrum of pediatric liver disease using a large sample population in Hubei Province, China.

## Materials and methods

### Patient selection and study design

**Figure 1:**
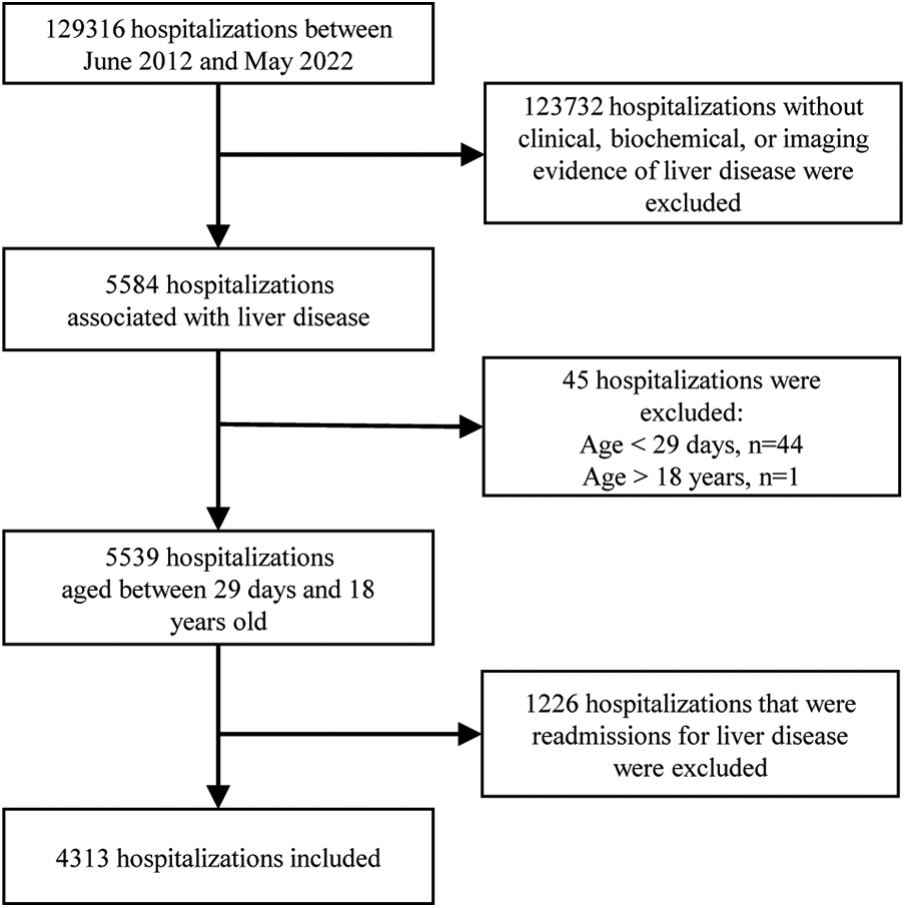
Flowchart of the enrollment of children with liver disease.

Participant information was acquired from medical records. Some of the missing information was collected through follow-up visits to outpatients or via the telephone. The data was extracted by reading the discharge diagnoses, which were categorized according to the 9th/10th revision of the International Classification of Diseases. Subsequently, each medical record was thoroughly reviewed to exclude cases that did not meet the inclusion criteria. Two experienced pediatric hepatologists determined the cause of the disease based on current opinions or guidelines. The participants were eligible if they met the following criteria: (i) they were children admitted to the pediatric ward, pediatric surgical ward, or pediatric intensive care unit (ICU); (ii) they had clinical, biochemical, or imaging evidence of liver disease, which may present as chronic liver disease, an acute hepatic event, or liver damage involved in systemic disorders; iii) they were aged between 29 days and 18 years old. Exclusion criteria included the absence of liver disease-related manifestations such as jaundice, abnormal liver function tests, hepatomegaly/hepatosplenomegaly, or abnormal liver imaging, as well as re-hospitalization for liver disease. Duplicate cases were excluded, meaning that only the medical record from the initial hospitalization for liver disease was considered, even if the patient was hospitalized multiple times (Figure 1).

The clinical data we collected included demographic information, medical history, physical examination results, laboratory test results, genetic test results, and treatment outcomes. Disease outcome relied on the patient's condition during hospital discharge. Regarding the relevant criteria of the World Health Organization, “malnutrition” in this study refers to a *z*-score of weight-for-stature less than −2 SD. Cholestasis is defined as the presence of (i) serum conjugated bilirubin >17.1 μmol/L when total bilirubin is <85.5 μmol/L, or (ii) a conjugated component >20% of the total when total bilirubin is >85.5 μmol/L (11, 12). Diagnosis of cirrhosis is based on liver histology or clinical and imaging findings (13). The following criteria are used to define acute liver failure: (i) acute onset of liver illness without signs of chronic liver disease; (ii) biochemical evidence of severe liver injury; and (iii) coagulopathy that is not improved by vitamin K. Prothrombin time (PT) ≥15 s or international normalized ratio (INR) ≥1.5 in the presence of hepatic encephalopathy (HE) or PT ≥ 20 s or INR ≥ 2.0 in the absence of HE (14).

Patients were categorized into five age groups, namely: 0–1 years, 1–3 years, 3–6 years, 6–12 years, and 12–18 years. To determine the etiological distribution, we divided the study population into fourteen sections: infection, biliary obstructive disease, inherited metabolic liver disease (IMLD), non-alcoholic fatty liver disease (NAFLD), autoimmune liver disease, drug-induced liver injury, poison-induced liver injury, parenteral nutrition-associated liver disease, vascular/ischemic liver disease, malignant liver mass, benign liver mass, rheumatologic disease, endocrine disease, and undiagnosed cases.

### Laboratory assessment

A laboratory assessment protocol was developed for children with liver disease based on a comprehensive physical examination and accurate clinical history. Examination measurements were conducted to assess a range of parameters including hematology, chemistry, microbiology, immunology, imaging, endoscopy, and histopathology. The selection of tests varied depending on the patient's age, clinical presentation, and the presence or absence of jaundice. Table 1 displays the laboratory methods used in our study.

**Table 1:** Laboratory assessment protocol for children with liver disease.

Investigations	Items
Laboratory tests (choose accordingly based on clinical cues)	
Initial work-up	General: aspartate aminotransferase, alanine aminotransferase, bilirubin, γ -glutamyl-transpeptidase, alkaline phosphatase, total protein, albumin, and serum bile acids, international normalized ratio, prothrombin time, partial thromboplastin time, urea, creatinine, sodium, potassium, calcium, phosphate, bicarbonate, creatine kinase, full blood count with differential, reticulocyte count, C-reactive protein, urine analysis (pH, glucose, ketones, and protein). Infectious: serology or PCR for cytomegalovirus, Epstein–Barr virus, hepatitis viruses A, B, C, D, and E, rubella virus, parvovirus B19, herpes simplex virus, toxoplasma, syphilis, human immunodeficiency virus; bacterial cultures of blood, urine, and other fluids; urine cytomegalovirus culture; direct immunofluorescence of swabs or tissue Metabolic: cholesterol, triglyceride, high-density lipoprotein cholesterol, low-density lipoprotein cholesterol, glucose, ammonia, blood gas, lactic acid, pyruvate, alpha-fetoprotein, amylase, lipase, copper concentration, 24-h urinary copper, serum iron, ferritin, transferrin, free thyroxine, free triiodothyronine, thyrotropin, cortisol, adrenocorticotropic hormone, alpha-1 antitrypsin Autoantibody spectrum: antinuclear antibodies, anti-smooth muscle antibodies, anti-liver kidney microsomal antibody type 1, anti-liver cytosol antibodies, anti-soluble liver antigen, anti-neutrophil cytoplasmic antibodies, anti-tissue transglutaminase, antiplatelet antibodies, complement, and immunoglobulins Others: serum matrix metalloproteinase-7; duodenal fluid examination; Vitamins A, D, and E
Second-line work-up	Infectious: serology or PCR for varicella-zoster virus, adenovirus, measles virus, novel bunyavirus, mycobacterium, Yellow fever virus, Dengue virus, Coxiella bunerii, plasmodium, spirochetes, parasite, fungus Metabolic: plasma and urinary amino acids; plasma acylcarnitine profile; urinary organic acids; plasma very long-chain fatty acids; enzyme activity in dried blood spots, leukocytes, or fibroblasts; blood galactose; sweat chloride analysis Others: blood and urine tests for toxins
Imaging investigations (If necessary)	Abdominal ultrasound, echocardiogram, electroencephalogram, x-ray, computed tomography, radionuclide hepatobiliary dynamic imaging, magnetic resonance cholangiopancreatography, endoscopic retrograde cholangiopancreatography, intraoperative cholangiography, eye visit (fundus oculi, and slit lamp examination)
Pathological examinations (If necessary)	Liver biopsy: routine histopathology, electron microscopy, immunohistochemistry, microbiology Bone marrow aspiration Skin Biopsy

### Genetic sequencing

If clinical, biochemical, or pathology features highly suggest a specific genetic disease, medical practitioners may decide to conduct genetic tests after obtaining the patient or guardian's informed consent. From 2012 to 2014, Sanger sequencing was the dominant method of analysis at our center. However, starting in 2015, there has been a gradual shift towards NGS as the primary test. NGS includes panel-based NGS, clinical exome sequencing (CES), and WES. When selecting Sanger sequencing, we usually performed a single-gene test based on clinical features due to limited resources, particularly for strong phenotypes: *SLC25A13* for citrin deficiency; *JAG1* and *NOTCH2* for Alagille syndrome; *ATP8B1*, *ABCB11*, and *ABCB4* for phenotypic progressive familial intrahepatic cholestasis; *HSD3B7* and *AKR1D1* for phenotypic congenital bile acid synthesis defect; *ATP7B* for Wilson disease; *UGT1A1* for unconjugated hyperbilirubinemia. Generally, patients with liver-focused disease phenotypes received panel-based NGS testing, while those with a wider range of disease phenotypes underwent CES or WES testing.

The genomic DNA of the patients and their parents was extracted from peripheral blood samples. The DNA fragments were amplified via polymerase chain reaction (PCR) and then hybridized with capture probes using the Agilent SureSelectXT2 Target Enrichment System (Agilent, Santa Clara, USA). The Illumina platform was used for sequencing in the genetic screening process. The human reference genome (hg19) was used to map the sequencing data, and the pathogenicity of the variations was categorized by the American College of Medical Genetics and Genomics recommendations (15). Sanger sequencing was used to validate all potential pathogenic variants, and quantitative real-time polymerase chain reaction (qPCR) was used to validate copy number variations (CNVs). Studies on cosegregation within the households were conducted. A positive genetic diagnosis was obtained for autosomal-recessive disease through the detection of two allelic genetic variations and for autosomal-dominant disease through the detection of one allelic genetic variation (15).

### Statistical analysis

Data processing and analysis were conducted using SPSS 23.0. Continuous variables were expressed as either the mean ± standard deviation or the median and interquartile range, following an assessment of the normality of the distribution. The chi-square or Fisher's exact test was employed to compare counting data. When necessary, the Mann-Whitney *U* test or an independent *t*-test was used to assess comparisons in different groups. We calculated the overall number of eligible admissions for each year between 2012 and 2022, categorized by age groups and the specific causes of liver disease. To determine the percentage of admissions by etiology and age group for each year, we conducted comprehensive analyses. We performed time trend analyses using linear regression with each study year as a dependent variable.

This study was reviewed and approved by the Medical Ethics Committee of Tongji Hospital, Tongji Medical College, Huazhong University of Science and Technology (TJ-IRB20220607).

## Results

### Overall distribution characteristics

Medical records of 4,313 patients were used for statistical analysis in this study. The clinical characteristics of this cohort are summarized in Table 2. The median age was 0.7 (0.2–4.5) years (range: 0.1–17.1 years). The largest number of patients in the study (54.5%) were aged 0–1 years. The rest were split between the age groups of 1–3 years (13.2%), 3–6 years (12.6%), 6–12 years (15.6%), and 12–18 years (4.1%). Males made up 58.5% of the participants. 90.8% of patients were referred to our hospital within six months of onset. The chief complaints included jaundice (1,922 cases), abnormal liver function tests (2,018 cases), abnormal liver imaging (218 cases), and organomegaly (155 cases). Upon admission, medical evaluations revealed that 15.5% of the children recruited suffered from malnutrition.

**Table 2:** Epidemiological characteristics of patients in different age groups.

Patient characteristics	Overall (*n* = 4,313)	0–1 years (*n* = 2,351)	1–3 years (*n* = 570)	3–6 years (*n* = 543)	6–12 years (*n* = 673)	12–18 years (*n* = 176)	*P*
Age [year, median (IQR)]	0.7 (0.2–4.5)	0.2 (0.2–0.3)	1.8 (1.3–2.3)	4.3 (3.6–5.2)	8.6 (7.3–10.3)	13.0 (12.4–13.8)	<0.001*
Gender (*n*, %)							0.010**
Male	2,523 (58.5)	1,405 (59.8)	312 (54.7)	301 (55.4)	386 (57.4)	119 (67.6)	
Female	1,790 (41.5)	946 (40.2)	258 (45.3)	242 (44.6)	287 (42.6)	57 (32.4)	
Admission time after onset (n, %)							<0.001**
0–6 months	3,917 (90.8)	2,267 (96.4)	478 (83.9)	475 (87.5)	563 (83.7)	134 (76.1)	
>6 months	396 (9.2)	84 (3.6)	92 (16.1)	68 (12.5)	110 (16.3)	42 (23.9)	
Cholestasis (*n*, %)	1,837 (42.6)	1,525 (64.9)	108 (18.9)	75 (13.8)	105 (15.6)	24 (13.6)	<0.001**
Cirrhosis (*n*, %)	334 (7.7)	195 (8.3)	23 (4.0)	21 (3.9)	60 (8.9)	35 (19.9)	<0.001**
Acute liver failure (*n*, %)	94 (2.2)	33 (1.4)	16 (2.8)	9 (1.7)	29 (4.3)	7 (4.0)	<0.001**
Malnutrition (*n*, %)	667 (15.5)	543 (23.1)	41 (7.2)	30 (5.5)	35 (5.2)	18 (10.2)	<0.001**
Etiology (*n*, %)							<0.001***
Infection	1,299 (30.1)	426 (17.3)	233 (40.9)	306 (56.4)	301 (44.7)	33 (18.8)	
Undiagnosed cases	1,111 (25.8)	890 (36.0)	95 (16.7)	64 (11.8)	52 (7.7)	10 (5.7)	
Biliary obstructive disease	686 (15.9)	545 (22.1)	67 (11.8)	32 (5.9)	33 (4.9)	9 (5.1)	
Inherited metabolic liver disease	599 (13.9)	274 (11.1)	86 (15.1)	79 (14.5)	110 (16.3)	50 (28.4)	
Non-alcoholic fatty liver disease	139 (3.2)	0 (0.0)	0 (0.0)	0 (0.0)	88 (13.1)	51 (29.0)	
Drug-induced liver injury	108 (2.5)	27 (1.1)	23 (4.0)	20 (3.7)	32 (4.8)	6 (3.4)	
Parenteral nutrition-associated liver disease	103 (2.4)	103 (4.2)	0 (0.0)	0 (0.0)	0 (0.0)	0 (0.0)	
Malignant liver mass	83 (1.9)	24 (1.0)	33 (5.8)	17 (3.1)	8 (1.2)	1 (0.6)	
Autoimmune liver disease	40 (0.9)	8 (0.3)	8 (1.4)	8 (1.5)	13 (1.9)	3 (1.7)	
Rheumatologic disease	36 (0.8)	4 (0.2)	12 (2.1)	4 (0.7)	11 (1.6)	5 (2.8)	
Benign liver mass	34 (0.8)	20 (0.8)	3 (0.5)	4 (0.7)	7 (1.0)	0 (0.0)	
Endocrine disease	32 (0.7)	23 (0.9)	1 (0.2)	1 (0.2)	5 (0.7)	2 (1.1)	
Poison-induced liver injury	23 (0.5)	2 (0.1)	7 (1.2)	5 (0.9)	7 (1.0)	2 (1.1)	
Vascular/ischemic liver disease	20 (0.5)	5 (0.2)	2 (0.4)	3 (0.6)	6 (0.9)	4 (2.3)	
ICU admission (*n*, %)	307 (7.1)	84 (3.4)	80 (14.0)	50 (9.2)	71 (10.5)	22 (12.5)	<0.001**
LOS [d, median(IQR)]	7.0 (5.0–13.0)	8.0 (5.0–14.0)	8.0 (5.0–15.0)	7.0 (5.0–11.0)	6.0 (4.0–11.0)	6.0 (3.0–10.0)	<0.001*
Outcome							0.021**
Death	50 (1.2)	17 (0.7)	7 (1.2)	8 (1.5)	15 (2.2)	3 (1.7)	
Survival	4,263 (98.8)	2,334 (99.3)	563 (98.8)	535 (98.5)	658 (97.8)	173 (98.3)	

ICU, intensive care unit; IQR, interquartile range; LOS, length of stay; Y, year.

* Kruskal-Wallis test.

** Chi-square test.

*** Fisher exact.

**Figure 2:**
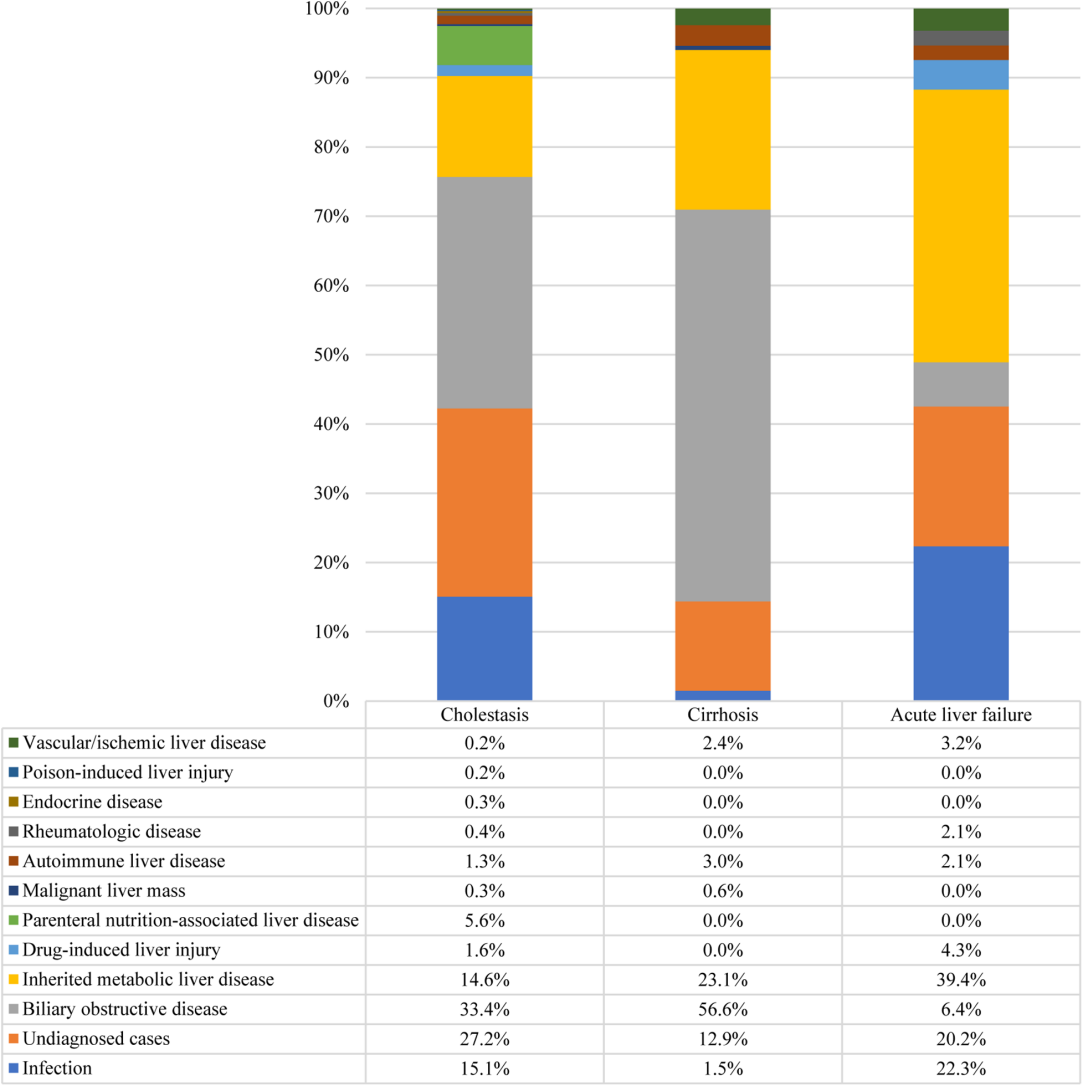
Distribution of causes of cholestasis, cirrhosis, and acute liver failure. There were 1,837 cases of cholestasis, 334 cases of cirrhosis, and 94 cases of acute liver failure.

**Figure 3:**
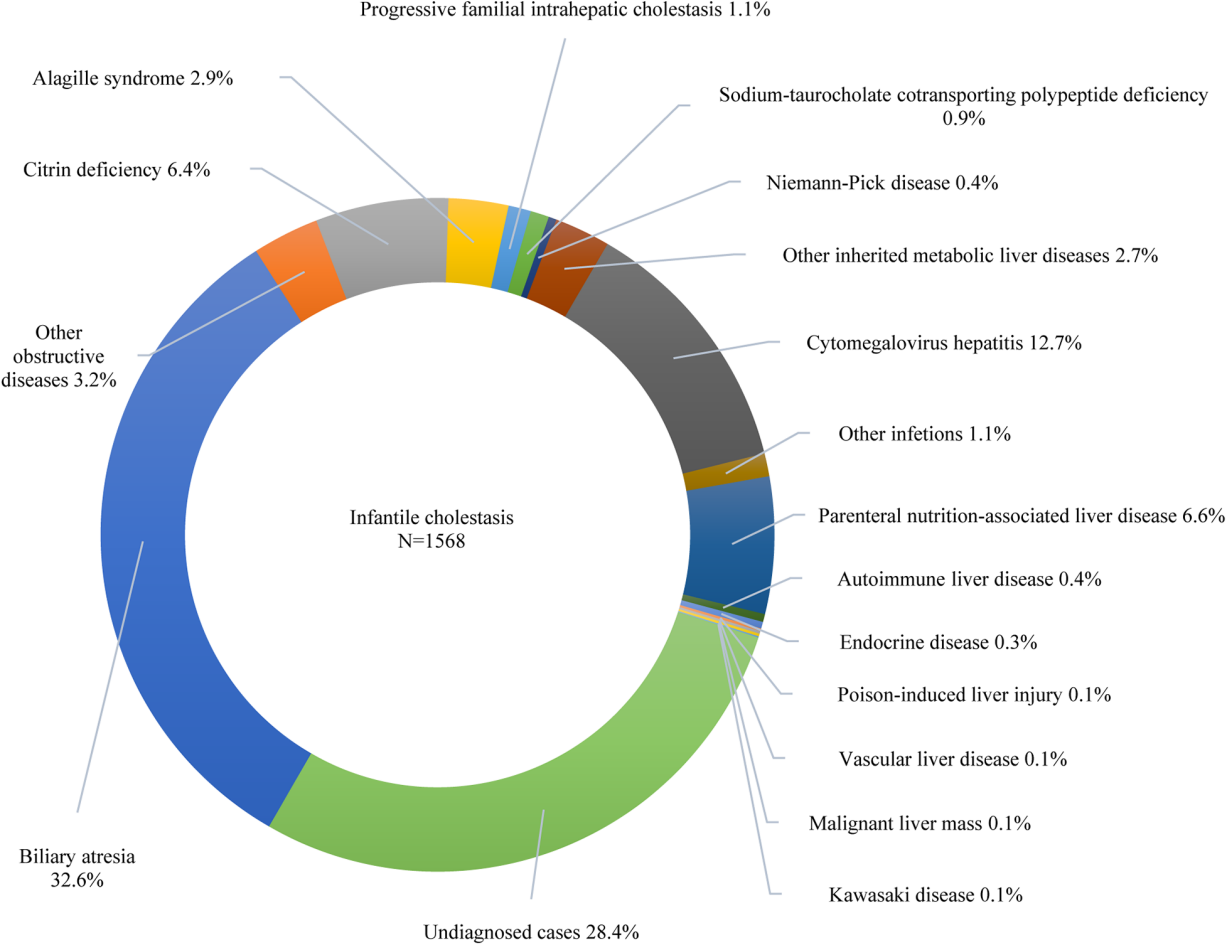
Distribution of causes of infantile cholestasis.

Furthermore, 42.6% of the participants exhibited cholestasis; pertinently, the 0–1 years age group had the highest incidence at 64.9%, followed by 1–3 years at 18.9%, 6–12 years at 15.6%, 3–6 years at 13.8%, and 12–18 years at 13.6% (*p* < 0.001). While 7.7% of the study participants presented with cirrhosis, acute liver failure was observed in 2.2% of the cohort. The etiologic composition of cholestasis, cirrhosis, and acute liver failure is shown in Figure 2. Infantile cholestasis accounted for a substantial percentage of pediatric liver disease, including 36.4% (1,568/4,313) of cases in this population, and 85.4% of childhood cholestasis. Figure 3 shows the distribution of causes of infantile cholestasis. Differences in the diagnostic yield among age groups were observed, with the highest rate of diagnosis (94.3%) found in children aged 12–18 years and the lowest rate (62.1%) in the age group of 0–1 years. Among the 4,313 admitted patients, 50 died, corresponding to a mortality rate of 1.2%. Furthermore, 7.1% of the patients needed ICU admission. The median length of stay for this cohort was 7.0 (5.0–13.0) days.

### Distributional characteristics by etiology

Infection was found to be the primary causative agent accounting for 30.1% (1,299/4,313) of cases in this cohort. The distribution of pathogens resulted in 1,192 cases of viruses, 70 cases of bacteria, 27 cases of sepsis, seven cases of fungus, and three cases of parasites. Epstein-Barr virus (EBV) hepatitis was the most common cause of infectious liver disease, with 760 reported cases. The median age of affected patients was 4.7 (2.7–7.3) years, with 419 males and 341 females. Cytomegalovirus (CMV) hepatitis (347 cases) ranked second among the causes of infectious diseases, with 204 cases in males and 143 in females. The median age of affected individuals was 0.2 (0.2–0.3) years, with 199 cases presenting as infantile cholestasis, 146 cases with elevated aminotransferases, and two cases with hepatomegaly. The remaining pathogens are detailed in Table 3. Notably, 21 patients presented with acute liver failure. The leading causes were sepsis (11 cases), EBV infection (seven cases), non-A-E virus infection (two cases), and bacterial infection (one case). In addition, cirrhosis was seen in five children with hepatitis B. In this group of patients with infectious liver disease, 18 patients died during hospitalization, resulting in a mortality rate of 1.4%. The causes of death were diverse and included sepsis (eight cases), EBV infection (three cases), and infections caused by non-A-E virus, enterovirus, novel bunyavirus, staphylococcus aureus, klebsiella pneumoniae, acinetobacter baumannii, and candida albicans (one case each).

**Table 3:** Clinical spectrum of non-hereditary liver diseases (*n* = 2,603).

Group	Age (year)	Gender (M/F)	Cause (number)
Infection (1,299)	2.9 (0.4–6.2)	749/550	Virus (1,192): Epstein–Barr virus (760), Cytomegalovirus (347), Hepatitis B virus (48), Non-A-E virus (14), Hepatitis C virus (5), Enterovirus (3), Hepatitis A virus (2), Adenovirus (2), Herpes simplex virus (2), Varicella zoster virus (2), Measles virus (2), Novel bunyavirus (2), Hepatitis E virus (1), Parvovirus B19 (1), Rubella virus (1) Bacteria (70): Salmonella (16), Escherichia coli (12), Syphilis (8), Staphylococcus aureus (6), Klebsiella pneumoniae (6), Brucella (5), Staphylococcus species (4), Mycoplasma (3), Streptococcus pneumoniae (3), Haemophilus influenzae (3), Mycobacterium tuberculosis (1), Acinetobacter baumannii (1), Enterobacter cloacae (1), Proteus (1) Sepsis (27) Parasite (7): Trichinella spiral (3), Toxoplasma (2), Trematode (2) Fungus (3): Candida albicans (2), Histoplasma capsulatum (1)
Biliary obstructive disease (686)	0.2 (0.1–0.6)	327/359	Biliary atresia (511), Biliary dilation (139), Gallstones (18), Biliary tract developmental abnormalities (6), Caroli disease (5), Bile duct perforation (3), Biliary ascariasis (2), Biliary hyperviscosity syndrome (1), Bile duct mass (1)
Non-alcoholic fatty liver disease (139)	11.3 (10.4–12.6)	118/21	Non-alcoholic fatty liver (76), Non-alcoholic steatohepatitis (63)
Drug-induced liver injury (108)	3.6 (1.0–7.7)	59/49	Acetaminophen (28), Herbal and dietary supplements (26), Anti-neoplastic drugs (21), Anti-infection drugs (19), Non-steroidal anti-inflammatory drugs (11), Antiepileptic drugs (2), Immunosuppressants (1)
Parenteral nutrition-associated liver disease (103)	0.2 (0.2–0.3)	73/30	Parenteral nutrition-associated liver disease (103)
Malignant liver mass (83)	2.2 (0.8–3.6)	51/32	Hepatoblastoma (66), Hepatocellular carcinoma (10), Hepatic sarcoma (1), Yolk sac tumor (1), Teratoma (1), Metastatic liver tumor (4)
Autoimmune liver disease (40)	3.9 (1.4–8.2)	20/20	Autoimmune hepatitis (29), Primary sclerosing cholangitis (6), Autoimmune sclerosing cholangitis (5)
Rheumatologic disease (36)	4.6 (1.6–7.1)	14/22	Juvenile idiopathic arthritis (26), Kawasaki disease (8), Systemic lupus erythematosus (2)
Benign liver mass (34)	0.4 (0.2–4.6)	13/21	Liver hemangioma (22), Dysplastic nodules (4), Liver cyst (3), Mesenchymal hamartoma of the liver (2), Focal nodular hyperplasia (2), Hepatic angiomyolipoma (1)
Endocrine disease (32)	0.3 (0.1–2.5)	22/10	Adrenal insufficiency (21), Congenital hypothyroidism (11)
Poison-induced liver injury (23)	4.2 (1.6–9.5)	13/10	Bee venom (8), Carbon monoxide (5), Rodenticide (3), Toxic chemical (3), Pesticide (1), Snake venom (1), Fish bile (1)
Vascular/ischemic liver disease (20)	6.3 (1.2–11.5)	11/9	Portal vascular malformation (16), Ischemic hepatitis (3), Hepatic sinusoidal obstruction syndrome (1)

F, female; M, male; Y, year.

Among the 1,111 cases without a definitive diagnosis, 890 (80.1%) involved infants. The median age was 0.3 (0.2–0.7) years. There were 683 males and 428 females. Of these, 567 cases exhibited abnormal liver function tests, 499 cases presented with cholestatic jaundice, 42 cases presented with organomegaly, and three cases showed abnormalities found on ultrasound. In addition, 43 cases presented with cryptogenic cirrhosis and 19 with acute liver failure. Genetic testing was performed on 35.3% (392/1,111) of cases and liver biopsy was performed on 17.1% (190/1,111) of cases. Specifically, 125 patients underwent both genetic testing and histological examinations, while 267 underwent only genetic testing, and 65 underwent only histological examinations. During hospitalization, there were 11 mortalities (1.0%), 66 patients showed no improvement (5.9%), and 1,034 patients (93.1%) showed improvement.

Biliary obstructive diseases, mainly consisting of biliary atresia and biliary dilatation, accounted for 15.9% (686/4,313) of the cases. Biliary atresia accounted for 32.6% of infantile cholestasis, with 511 reported cases. The median age of cases with biliary atresia was 0.2 (0.1–0.2) years, with 269 males and 242 females. Biliary dilation ranked second among obstructive causes, with 139 reported cases. The affected individuals had a median age of 1.9 (0.6–3.7) years, with 41 males and 98 females. There were 88 cases of biliary dilatation with cholestasis and 51 with elevated transaminases. The remaining causes are detailed in Table 3. In this group of patients, two children with biliary atresia and four with biliary dilation experienced acute liver failure. Additionally, cirrhosis was observed in 180 children with biliary atresia, six with biliary dilation, two with Caroli disease, and one with biliary tract developmental abnormalities. A total of four fatalities were recorded due to biliary atresia during hospitalization, resulting in a mortality rate of 0.6%.

IMLDs represented 13.9% (599/4,313) of the study population, with sixty diverse forms detected. The median age was 1.5 (0.3–6.6) years. The analysis included 370 males and 229 females. Distribution characteristics according to pathogenesis and age of onset are shown in Tables 4, 5, respectively. In this cohort, 54.4% of cases were initiated during infancy and 80.5% of cases progressed before the age of six years. Wilson disease, citrin deficiency, glycogen storage disease, and Alagille syndrome were the most common IMLDs, accounting for 66.4% of cases. In 154 cases of Wilson disease, the median age was 8.6 (5.4–11.3) years, with a higher proportion of male patients (65.6%). The clinical signs of liver involvement ranged from incidental observations of aberrant liver function to severe hepatic failure. Citrin deficiency was the second most common genetic cause in the cohort with 101 cases. There were 58 males and 43 females, with a median age of 0.2 (0.2–0.3) years, and 99.0% of cases presented with infantile cholestasis. Glycogen storage disease (92 cases) was typically identified through liver enlargement or elevated aminotransferases. The median age was 2.5 (1.5–6.0) years, and there was a male predominance (66.3%). Out of the 51 cases of Alagille syndrome, 29 were male and 22 were female, with a median age of 0.2 (0.2–0.6) years. The majority of patients (88.2%) presented with infantile cholestasis and multisystem involvement. Other infrequent IMLDs presented diverse clinical manifestations. In this group, 37 cases presented with acute liver failure. The cases included 18 cases of Wilson disease, 10 cases of citrin deficiency, three cases of tyrosinemia, two cases of infantile liver failure syndrome, and one case each of ornithine transcarbamylase deficiency, dicarboxylic aminoaciduria, congenital disorder of glycosylation, and Alagille syndrome. In addition, 77 cases of cirrhosis were identified with causes including Wilson disease (55 cases), citrin deficiency (three cases), polycystic kidney disease 4 (three cases), glycogen storage disease (two cases), progressive familial intrahepatic cholestasis (two cases), Niemann-Pick disease (two cases), and cystic fibrosis liver disease (two cases). There was one case each of congenital bile acid synthesis defect, Alstrom syndrome, nephronophthisis 12, Shwachman-Diamond syndrome, immunodeficiency 28 with mycobacteriosis, Adams-Oliver syndrome, familial visceral amyloidosis, and hereditary hemorrhagic telangiectasia syndrome. In this group of patients, one individual died from ornithine transcarbamylase deficiency and four died from Wilson disease, resulting in a mortality rate of 0.8%.

**Table 4:** Clinical spectrum of inherited metabolic liver diseases (*n* = 599).

Group	Cause (number)
Disorders of metals (154)	Wilson disease (154)
Disorders of amino acid metabolism (117)	Citrin deficiency (101), Ornithine transcarbamylase deficiency (5), Carbamoyl phosphate synthetase 1 deficiency (2), Dicarboxylic aminoaciduria (4), Tyrosinemia (3), Methylmalonic acidemia (1), Propionic acidemia (1)
Disorders of carbohydrate metabolism (93)	Glycogen storage disease (92), Congenital disorder of glycosylation (1)
Disorders of bile acid metabolism (55)	Progressive familial intrahepatic cholestasis (21), Sodium-taurocholate cotransporting polypeptide deficiency (29), Congenital bile acid synthesis defect (5)
Inherited cholangiopathies (54)	Alagille syndrome (51), Cystic fibrosis liver disease (3)
Disorders of tetrapyrrole metabolism (38)	UDP-glucuronosyltransferase A1 deficiency (29), Dubin-Johnson syndrome (5), Rotor syndrome (3), Hereditary coproporphyria (1)
Lysosomal storage diseases (31)	Niemann-Pick disease (20), Mucopolysaccharidosis (6), Gaucher disease (4), Lysosomal acid lipase deficiency (1)
Ciliopathy (13)	Polycystic kidney disease 4, with or without hepatic disease (6), Alstrom syndrome (2), Polycystic kidney disease 1 (2), Polycystic kidney disease 2 (1), Nephronophthisis 12 (1), Renal-hepatic-pancreatic dysplasia 1 (1)
Disorders of lipid and lipoprotein metabolism (10)	Reynolds syndrome (4), Combined hyperlipidemia, familial (2), Congenital generalized lipodystrophy type 2 (1), Sitosterolemia due to ABCG8 deficiency (1), Hyperalphalipoproteinemia (1), Hypobetalipoproteinemia (1)
Disorders of nucleobase, nucleotide and nucleic acid metabolism (6)	Shwachman-Diamond syndrome (5), Aicardi-Goutieres syndrome 1 (1)
Immunodeficiency (4)	Immunodeficiency 28, mycobacteriosis (1), Immunodeficiency 31C, chronic mucocutaneous candidiasis, autosomal dominant (1), Immunodeficiency, X-linked, with hyper-IgM (1), Lymphoproliferative syndrome, X-linked, 2 (1)
Disorders of fatty acid and ketone body metabolism (3)	Primary carnitine deficiency (2), Mitochondrial 3-hydroxy-3-methylglutaryl-CoA synthase deficiency (1)
Mitochondrial diseases (3)	Transient infantile hypertriglyceridemia (2), Mitochondrial DNA depletion syndrome (1)
Chromosomal diseases (3)	Down syndrome (2), Turner syndrome (1)
Others (15)	Infantile liver failure syndrome (3), Zellweger syndrome (1), Hermansky-Pudlak syndrome 10 (1), Adams-Oliver syndrome (1), Hepatic fibrinogen storage disease (1), Familial visceral amyloidosis (1), Kabuki syndrome 1 (1), Kleefstra syndrome 2 (1), XFE progeroid syndrome (1), Hereditary hemorrhagic telangiectasia syndrome (1), Autoinflammation, immune dysregulation, and eosinophilia (1), Diabetes mellitus, neonatal, with congenital hypothyroidism (1), Noonan syndrome 6 (1)

**Table 5:** Age distribution characteristics of inherited metabolic liver diseases (*n* = 599).

Age at onset	Cause (number)
0–1 years (44 kinds, 326 cases)	Citrin deficiency (100), Alagille syndrome (47), Glycogen storage disease (32), Sodium-taurocholate cotransporting polypeptide deficiency (28), UDP-glucuronosyltransferase A1 deficiency (22), Progressive familial intrahepatic cholestasis (18), Niemann-Pick disease (14), Congenital bile acid synthesis defect (4), Shwachman-Diamond syndrome (4), Dicarboxylic aminoaciduria (4), Polycystic kidney disease 4, with or without hepatic disease (4), Dubin-Johnson syndrome (3), Rotor syndrome (3), Tyrosinemia (3), Ornithine transcarbamylase deficiency (3), Reynolds syndrome (3), Infantile liver failure syndrome (2), Transient infantile hypertriglyceridemia (2), Combined hyperlipidemia, familial (2), Polycystic kidney disease 1 (2), Wilson disease (2), Down syndrome (2), Methylmalonic acidemia (1), Propionic acidemia (1), Carbamoyl phosphate synthetase 1 deficiency (1), Congenital disorder of glycosylation (1), Cystic fibrosis liver disease (1), Gaucher disease (1), Renal-hepatic-pancreatic dysplasia 1 (1), Polycystic kidney disease 2 (1), Sitosterolemia due to ABCG8 deficiency (1), Congenital generalized lipodystrophy type 2 (1), Hypobetalipoproteinemia (1), Aicardi-Goutieres syndrome 1 (1), Lymphoproliferative syndrome, X-linked, 2 (1), Primary carnitine deficiency (1), Mitochondrial 3-hydroxy-3-methylglutaryl-CoA synthase deficiency (1), Mitochondrial DNA depletion syndrome (1), Turner syndrome (1), Hermansky-Pudlak syndrome 10 (1), Zellweger syndrome (1), Kleefstra syndrome 2 (1), Kabuki syndrome 1 (1), Autoinflammation, immune dysregulation, and eosinophilia (1)
1–3 years (20 kinds, 81 cases)	Glycogen storage disease (43), Wilson disease (11), Mucopolysaccharidosis (4), Gaucher disease (3), Ornithine transcarbamylase deficiency (3), Alagille syndrome (2), Progressive familial intrahepatic cholestasis (2), Niemann-Pick disease (2), Citrin deficiency (1), UDP-glucuronosyltransferase A1 deficiency (1), Dubin-Johnson syndrome (1), Cystic fibrosis liver disease (1), Lysosomal acid lipase deficiency (1), Hepatic fibrinogen storage disease (1), Shwachman-Diamond syndrome (1), Primary carnitine deficiency (1), Alstrom syndrome (1), Adams-Oliver syndrome (1), Noonan syndrome 6 (1), Immunodeficiency 31C, chronic mucocutaneous candidiasis, autosomal dominant (1)
3–6 years (16 kinds, 75 cases)	Wilson disease (42), Glycogen storage disease (14), UDP-glucuronosyltransferase A1 deficiency (3), Alagille syndrome (2), Mucopolysaccharidosis (2), Polycystic kidney disease 4, with or without hepatic disease (2), Nephronophthisis 12 (1), Sodium-taurocholate cotransporting polypeptide deficiency (1), Congenital bile acid synthesis defect (1), Infantile liver failure syndrome (1), Niemann-Pick disease (1), Carbamoyl phosphate synthetase 1 deficiency (1), Familial visceral amyloidosis (1), XFE progeroid syndrome (1), Hereditary coproporphyria (1), Diabetes mellitus, neonatal, with congenital hypothyroidism (1)
6–12 years (9 kinds, 84 cases)	Wilson disease (73), Niemann-Pick disease (3), Glycogen storage disease (2), Cystic fibrosis liver disease (1), Progressive familial intrahepatic cholestasis (1), UDP-glucuronosyltransferase A1 deficiency (1), Hyperalphalipoproteinemia (1), Reynolds syndrome (1), Immunodeficiency 28, mycobacteriosis (1)
12–18 years (7 kinds, 33 cases)	Wilson disease (26), UDP-glucuronosyltransferase A1 deficiency (2), Dubin-Johnson syndrome (1), Glycogen storage disease (1), Hereditary hemorrhagic telangiectasia syndrome (1), Alstrom syndrome (1), Immunodeficiency, X-linked, with hyper-IgM (1)

NAFLD accounted for 3.2% (139/4,313) of the total cases, ranking fifth in the group. The median age was 11.3 (10.4–12.6) years, with a male predominance (84.9%). Assessment of body mass index revealed that 137 individuals were classified as obese and two as overweight. Forty-nine patients exhibited insulin resistance, 24 presented with dyslipidemia, 10 were diagnosed with type 2 diabetes mellitus, and two had hypertension. Non-alcoholic fatty liver was diagnosed in 76 cases and non-alcoholic steatohepatitis in 63 cases.

The remaining etiologies are detailed in Table 3. Drug-induced liver injury, parenteral nutrition-associated liver disease, malignant liver mass, autoimmune liver disease, rheumatologic disease, benign liver mass, endocrine disease, poison-induced liver injury, and vascular/ischemic liver disease were, respectively, seen in 108 (2.5%), 103 (2.4%), 83 (1.9%), 40 (0.9%), 36 (0.8%), 34 (0.8%), 32 (0.7%), 23 (0.5%), 20 (0.5%) cases. Of note, the less common diseases, like drug-induced liver injury and autoimmune liver disease, were difficult to diagnose and required specialized attention. Out of the 108 cases of drug-induced liver disease, 10 were diagnosed more than six months after the onset of the disease, with the longest period being 12 months.

Table 6 presents the distribution of common diseases in different age groups. In our study, biliary atresia and CMV hepatitis were prevalent during infancy, whereas EBV-associated hepatitis was the most frequent in the age group 1–12 years. Moreover, NAFLD and Wilson disease were more common in the age group of 12–18 years.

**Table 6:** Top five causes among cases with a definite diagnosis.

Age group	Causes	Number (proportion)
0–1 years (*n* = 1,461)		
	Biliary atresia	493 (33.7%)
	Cytomegalovirus hepatitis	341 (23.3%)
	Parenteral nutrition-associated liver disease	103 (7.0%)
	Citrin deficiency	96 (6.6%)
	Alagille syndrome	44 (3.0%)
1–3 years (*n* = 475)		
	Epstein-Barr virus hepatitis	193 (40.6%)
	Biliary dilatation	47 (9.9%)
	Glycogen storage disease	37 (7.8%)
	Hepatoblastoma	31 (6.5%)
	Drug-induced liver injury	23 (4.8%)
3–6 years (*n* = 479)		
	Epstein-Barr virus hepatitis	273 (57.0%)
	Wilson disease	39 (8.1%)
	Biliary dilatation	23 (4.8%)
	Drug-induced liver injury	20 (4.2%)
	Glycogen storage disease	17 (3.5%)
6–12 years (*n* = 621)		
	Epstein-Barr virus hepatitis	245 (39.5%)
	Non-alcoholic fatty liver disease	88 (14.2%)
	Wilson disease	74 (11.9%)
	Drug-induced liver injury	32 (5.2%)
	Biliary dilatation	20 (3.2%)
12–18 years (*n* = 166)		
	Non-alcoholic fatty liver disease	51 (30.7%)
	Wilson disease	32 (19.3%)
	Epstein-Barr virus hepatitis	24 (14.5%)
	Glycogen storage disease	7 (4.2%)
	Drug-induced liver injury	6 (3.6%)

### Cases with genetic testing

**Figure 4:**
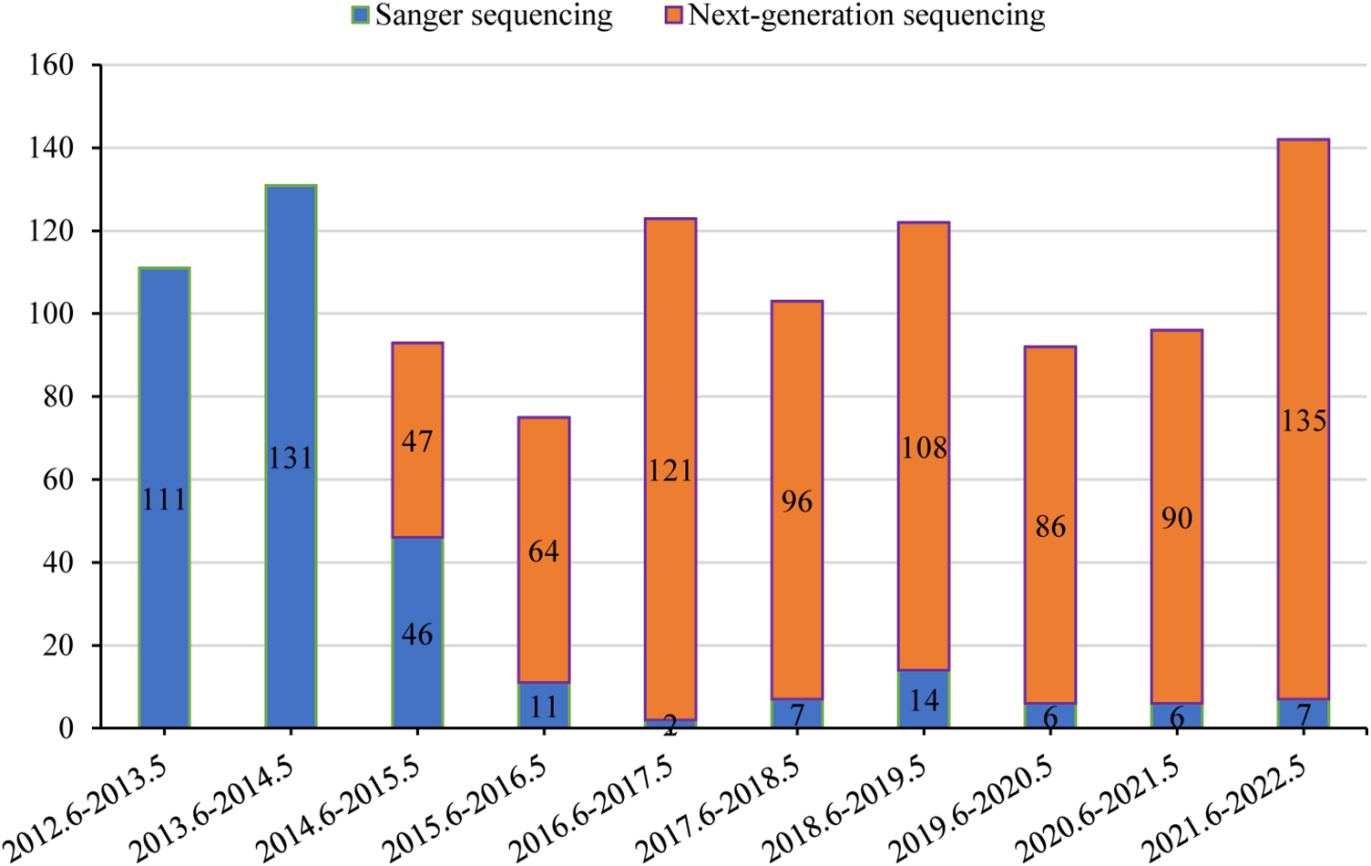
Temporal distribution of genetic testing.

Genetic results for 1,088 patients were collected from medical records. The temporal distribution of genetic testing is illustrated in Figure 4. Positive genetic diagnoses were established in 478/1,088 (43.9%) of patients, with Sanger sequencing detecting 122/341 cases and NGS identifying 356/747 cases. Out of the 610 cases in which genetic testing was inconclusive, 218 were later diagnosed with other clinical conditions. These included 76 cases of infectious liver diseases, 48 cases of biliary obstructive diseases, 31 cases of IMLDs, 31 cases of parenteral nutrition-associated liver disease, 15 cases of drug-induced liver injury, 13 cases of autoimmune liver disease, two cases of NAFLD, and two cases of portal vascular malformation.

Notably, 31 patients fulfilled the clinical diagnostic criteria but did not receive a conclusive genetic diagnosis. Clinically, 20 patients were diagnosed with citrin deficiency; however, genetic testing detected a heterozygous mutation in the *SLC25A13* gene (nine via Sanger sequencing and 11 via panel-based NGS). Genetic testing (two via Sanger sequencing, two via panel-based NGS, and one via WES) identified a heterozygous mutation in the *ATP7B* gene for five individuals clinically diagnosed with Wilson disease. In three cases of children clinically diagnosed with glycogen storage disease, NGS testing failed to identify the expected pathogenic variants (two via panel-based NGS and one via WES). Additionally, three individuals were clinically diagnosed with Alagille syndrome. No pathogenic variants were identified in the *JAG1* or *NOTCH2* gene through Sanger sequencing.

Moreover, there were still 392 cases without a definitive diagnosis. Out of these, 269 cases presented with cholestatic jaundice, 106 cases showed abnormal liver function tests, 15 cases presented with organomegaly, and two cases showed abnormalities detected on ultrasound.

### Time trends between June 2012 and May 2022

**Figure 5:**
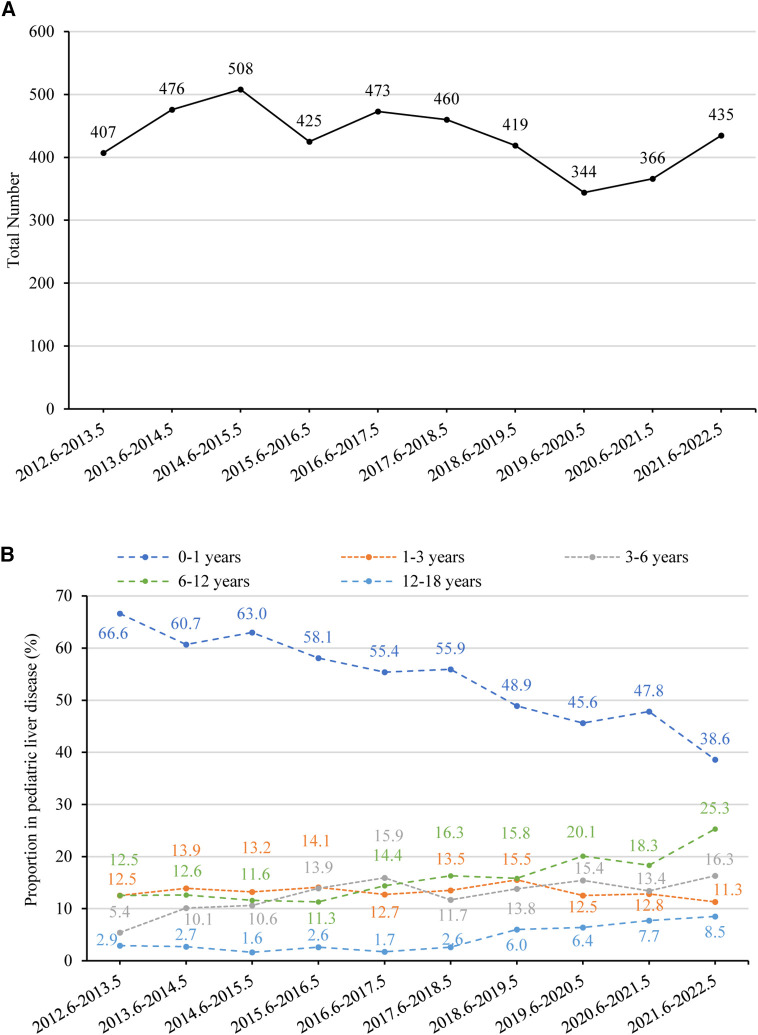
(**A**) number of pediatric liver disease-related admissions between June 2012 and May 2022. The time trend was assessed using linear regression analysis. The annual number of pediatric liver diseases remained stable (*p* = 0.145). (**B**) Percentage of children with liver disease in different age groups between June 2012 and May 2022. The proportion of children aged 0–1 decreased significantly (*p* < 0.001), while the proportion of those aged 1–3 remained stable (*p* = 0.444). There was an increasing trend in the proportion of children aged 3–6 (*p* = 0.008), 6–12 (*p* = 0.001), and 12–18 (*p* = 0.002).

Overall, the annual number of pediatric liver diseases remained stable between 2012 (407 cases) and 2022 (435 cases) (*p *= 0.145) (Figure 5A). The percentage of infants aged 0–1 with liver diseases significantly fell from 66.6% (*n* = 271) to 38.6% (*n* = 168) (*p *< 0.001). In contrast, there was a rising tendency in the percentage of children aged 3–18. There was a rise in the percentage of children belonging to the age range of 3–6 years, from 5.4% (*n* = 22) to 16.3% (*n* = 71) (*p *= 0.008), in the age range of 6–12 years, from 12.5% (*n* = 51) to 25.3% (*n* = 110) (*p *= 0.001), and in the age category of 12–18 years, from 2.9% (*n* = 12) to 8.5% (*n* = 37) (*p *= 0.002). The percentage of children aged 1–3 years remained relatively constant, declining from 12.5% (*n* = 51) to 11.3% (*n* = 49) (*p *= 0.444) (Figure 5B).

**Figure 6:**
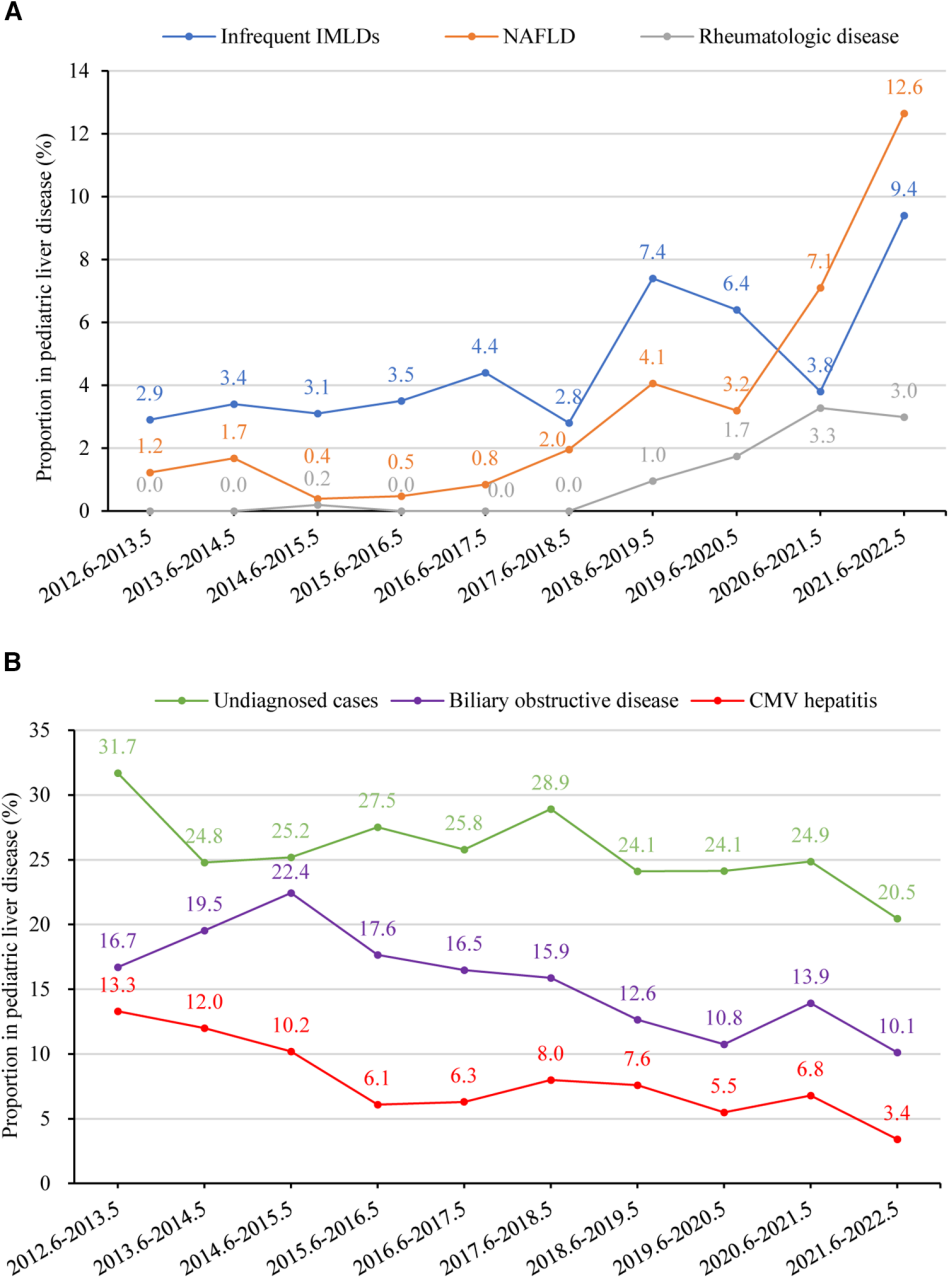
(**A**) percentage of children with liver disease in different etiology groups from 2012 to 2022 with a rising trend (*p* < 0.05). Time trends were assessed using linear regression analysis. The proportions of infrequent IMLDs, NAFLD, and rheumatologic disease showed an increasing trend (*p*-values 0.020, 0.006, and 0.002, respectively). (**B**) Percentage of children with liver disease in different etiology groups between June 2012 and May 2022 with a declining trend (*p* < 0.05). The proportions of undiagnosed cases, biliary obstructive disease, and CMV hepatitis showed a declining trend (*p*-values 0.031, 0.003, and 0.002, respectively). CMV, Cytomegalovirus; IMLD, inherited metabolic liver disease, infrequent IMLDs, defined as IMLDs excluding Wilson disease, citrin deficiency, glycogen storage disease, and Alagille syndrome. NAFLD, non-alcoholic fatty liver disease.

Figures 6A,B provide a summary of liver disease with rising and declining trends between 2012 and 2022 (*p *< 0.05), respectively. The percentage of undiagnosed cases declined from 31.7% (*n* = 129) to 20.5% (*n* = 89) (*p *= 0.031). In contrast, there was a significant upsurge in the percentage of NAFLD, rising from 1.2% (*n* = 5) to 12.6% (*n* = 55) (*p *= 0.006). Additionally, the percentage of rheumatologic disease showed an increasing trend from 0.0% (*n* = 0) to 3.0% (*n* = 13) between 2012 and 2022 (*p *= 0.002). Liver disease that significantly declined between 2012 and 2022 was biliary obstructive disease (from 16.7% to 10.1%, *p *= 0.003). The overall percentage of infectious liver diseases remained relatively stable, with fluctuations between 31.2% (*n* = 127) to 27.4% (*n* = 119) (*p *= 0.266). However, CMV hepatitis demonstrated a significant decline from 13.3% (*n* = 54) to 3.4% (*n* = 15) (*p *= 0.002). Furthermore, overall IMLDs presented a stable trend, shifting from 11.3% (*n* = 46) to 15.9% (*n* = 69) (*p *= 0.369); however, infrequent IMLDs (excluding Wilson disease, citrin deficiency, glycogen storage disease, and Alagille syndrome) displayed an increased trend from 2.9% (*n* = 12) to 9.4% (*n* = 41) (*p *= 0.020). Diseases related to liver injury with a relatively stable trend between 2012 and 2022 were drug-induced liver injury (from 1.2% to 1.8%, *p *= 0.170), parenteral nutrition-associated liver disease (from 3.4% to 2.8%, *p *= 0.596), malignant liver mass (from 0.5% to 2.5%, *p *= 0.278), autoimmune liver disease (from 1.5% to 1.1%, *p *= 0.521), benign liver mass (from 0.0% to 0.7%, *p *= 0.501), endocrine disease (from 0.5% to 0.5%, *p *= 0.724), poison-induced liver injury (from 0.2% to 0.9%, *p *= 0.764), and vascular/ ischemic liver disease (from 0.5% to 0.2%, *p *= 0.462).

The etiology of liver disease in infancy was analyzed for time trends, given that half of the cases in this cohort occurred during infancy (Table 7). There was a decreasing trend in the percentage of cytomegalovirus hepatitis among liver diseases in infancy (*p *= 0.023). However, the percentage of undiagnosed cases remained relatively stable (*p* = 0.147).

**Table 7:** Percentage of children aged 0–1 years with liver disease from 2012 to 2022.

Time	0–1 years (Total number)	Infection (Proportion)	Cytomegalovirus hepatitis (Proportion)	Biliary obstructive disease (Proportion)	Inherited metabolic liver disease (Proportion)	Parenteral nutrition- associated liver disease (Proportion)	Undiagnosed cases (Proportion)
2012.6–2013.5	271	25.5%	19.9%	21.8%	7.4%	5.2%	38.7%
2013.6–2014.5	289	24.2%	19.0%	26.3%	13.1%	3.8%	30.1%
2014.6–2015.5	320	20.6%	16.3%	27.5%	10.0%	3.1%	34.1%
2015.6–2016.5	247	11.7%	10.1%	24.3%	12.6%	4.9%	41.3%
2016.6–2017.5	262	15.6%	11.5%	22.1%	11.5%	5.0%	38.2%
2017.6–2018.5	257	17.5%	14.0%	23.0%	8.6%	2.7%	42.4%
2018.6–2019.5	205	17.1%	15.6%	20.5%	14.6%	2.9%	40.0%
2019.6–2020.5	157	15.3%	12.1%	17.2%	15.9%	5.1%	38.9%
2020.6–2021.5	175	15.4%	13.7%	26.3%	8.6%	5.7%	38.3%
2021.6–2022.5	168	11.9%	8.3%	17.9%	18.5%	7.1%	40.5%
*P*	-	0.011	0.023	0.134	0.123	0.227	0.147
Time trend	-	Decline	Decline	Stable	Stable	Stable	Stable

Time trends were assessed using linear regression analysis.

## Discussion

In this study, we described the epidemiological features in a large cohort of hospitalized children with liver disease in Hubei Province, China. Different age groups exhibit distinct characteristics. While infants typically display cholestatic jaundice, older children may exhibit abnormal liver function tests or organomegaly, in addition to jaundice (7). Liver disease in infancy makes up the largest group in pediatric liver disease. Even if the child does not display severe symptoms, an infant with liver disease ought to be treated as a medical emergency (8). Our study showed that over half of these patients experienced symptoms during infancy. This contradicts a survey conducted in California, USA, which revealed that half of all childhood liver disease occurs in the 16–21 years age group (16). These regional disparities contribute to this phenomenon. In addition, this study shows the lowest etiological diagnosis rate (62.1%) for cases in the 0–1 years age group. Cholestasis is a prevalent and severe condition in childhood (17). The incidence of cholestasis is believed to be 1:2,500 infants in North America (12). In our study population, cholestasis had a prevalence of 64.9% among infants with liver disease. Moreover, due to the young age and invasiveness, examination in infancy is limited. However, several of these maladies may have notable clinical consequences in the long run if they are not diagnosed early and treated promptly (8). Liver disease in infancy should therefore be of great clinical concern.

Infectious hepatitis may arise from a hepatotropic viral infection or as part of a systemic disorder. The symptoms can vary greatly, with a range of presentations including asymptomatic elevation in aminotransferases, cholestasis, acute liver failure, hepatic fibrosis, and cirrhosis (18). Consistent with prior research conducted in another region of China (19), infection remained the most common entity encountered, especially EBV and CMV infections in this study. CMV hepatitis is predominantly seen in young infants, whereas EBV hepatitis is predominantly seen in children over 1 year of age. The prevalence of hepatitis B virus infection in children was extremely low due to the widespread use of vaccinations (20). EBV is a worldwide widespread virus, infecting more than 90% of the world's population by maturity (21). The prevalence of EBV infection in Chinese children is more than 50% before the age of 3 years and more than 90% after the age of 8 years. In comparison, EBV infections occur at an earlier age than in the United States (22). In our cohort, the median age of patients with EBV hepatitis was 4.7 (2.7–7.3) years. Cases of acute liver failure and death were also reported. Adequate attention should also be paid to the most common form of EBV hepatitis in Chinese children.

CMV is a herpes virus with high (40%–100%) seroprevalence rates in adults (23). The incidence of CMV is negatively correlated with a country's level of socioeconomic development. It is more widespread in the Global South and Asia, whereas it is less frequent in Europe and the United States (24). In China, it was previously thought that CMV infection was the main cause of liver disease in infancy. This study also showed that CMV hepatitis was the second most common cause of liver disease in infancy and accounted for 12.7% of infantile cholestasis, behind biliary atresia and undiagnosed cases. However, it is encouraging that our study showed a downward trend in hospitalizations for cytomegalovirus hepatitis over the last decade. Over the past two decades, China has experienced significant demographic changes, including a decline in the birth rate from 14% to 6.8% and accelerated aging (25). In our cohort, the proportion of liver disease in infancy has also decreased. Furthermore, our study revealed a decreasing trend in the proportion of CMV hepatitis in liver disease during infancy. This suggests that demographic changes are not a significant influencing factor. This may be related to the increasing interest in CMV infection and the improved understanding of the etiology of pediatric liver disease. CMV infection is often observed in these cases, but it is not the primary cause of certain liver diseases (26). To our knowledge, no similar time-trend surveys on the proportion of CMV hepatitis among hospitalized children with liver disease in other centers in China have been conducted. Therefore, our findings require further validation through a national multicenter study.

About 20% of pediatric liver transplants are performed for single-gene liver diseases, and almost half of chronic liver diseases that manifest in childhood have a genetic basis (27). Clinical features that are similar and caused by IMLDs may be under-recognized or misdiagnosed due to their non-specific nature (4). This study observed that IMLD presents a range of clinical manifestations. Some cases only or mainly affect the liver, while others exhibit extrahepatic or systemic effects. With the growing popularity and reduced cost of genetic testing, early detection of liver disease is becoming more common (1, 28). In various genetic backgrounds, the spectrum of diseases differs extensively. The prevalent IMLDs in this group were Wilson disease, citrin deficiency, glycogen storage disease, and Alagille syndrome, which aligns with prior reported data (29). The prevalence of common inherited liver diseases varies by age. Citrin deficiency and Alagille syndrome were frequently observed in cases of infantile inherited cholestasis in our cohort. This finding is consistent with the results of a previous study conducted in Shanghai, China (30). Conversely, glycogen storage disease was more prevalent in toddlers and preschoolers with enlarged livers. Wilson disease, however, was more frequently observed in older children. Furthermore, our study showed that an increase in the percentage of infrequent or newly identified IMLDs was noted. For example, Sodium-taurocholate cotransporting polypeptide deficiency, a newly described inborn error of bile acid metabolism, was first reported in 2015 (31). Refractory hypercholanemia, brief cholestatic jaundice in infancy, and unconjugated hyperbilirubinemia in newborns are the primary clinical manifestations of this disease (32, 33). The identification of this condition has strengthened our comprehension of the reasons behind infantile cholestasis.

Biliary atresia is the foremost chronic liver condition afflicting children and responsible for most pediatric liver transplants worldwide. The incidence of it varies from 1 in 5,000 to 1 in 19,000 live births, with a higher prevalence in Asian nations than in European countries (12). In this investigation, biliary atresia emerged as the primary cause of liver diseases among infants, causing 32.6% of infantile cholestasis, consistent with prior research (12). Furthermore, it has been found that biliary atresia is the most frequent cause of liver cirrhosis, which is consistent with previous research (34). However, detecting biliary atresia in its early stages remains challenging, and further investigation is necessary.

NAFLD has become the leading cause of chronic liver disease in high-income nations due to the worldwide outbreak of obesity and overweight (35). While most patients have not exhibited obvious symptoms, pediatric NAFLD demonstrates a potential for decreased survival without liver transplantation when compared to their peers without the condition (36, 37). Anderson et al. reported that worldwide, the prevalence of NAFLD was 7.6% in children within the general population and 34% in obese pediatric populations (38). According to a systematic review, the prevalence of pediatric NAFLD has risen to 7.10%, from 4.42% before 2010 in the last decade in Asia (39). Our research indicates that the proportion of NAFLD cases in hospitalized children with liver disease has increased almost 10 times over the last decade. In addition, there has been a sharp rise in this rate from June 2020 to May 2022, which may be linked to the impact of the COVID-19 pandemic. Notably, a considerable portion of patients remain without diagnosis and treatment due to insufficient diagnostic tools and the absence of effective pharmaceutical remedies (40). Enhancing future care for children with NAFLD necessitates a superior understanding of the disease's natural progression and underlying pathology (41).

The etiological makeup of children admitted to our center with liver disease has undergone significant change in the past decade. Liver disease in patients with systemic disease is increasingly recognized. For example, an increase in the proportion of rheumatologic disease may be related to the severity of the disease and clinical considerations. In addition, there has been a significant decrease in the percentage of undiagnosed cases, from 31.7% to 20.5%. However, the etiological identification of pediatric liver disease remains a challenge, especially in developing countries. In our cohort, out of the 1,111 cases with an unknown diagnosis, only 35.3% underwent genetic testing and only 17.1% underwent liver biopsy, owing to limited resources. Furthermore, as 80.1% of the cases occurred during infancy, the restricted diagnostic options available, combined with vague or non-existent symptoms, make it challenging to determine the cause (4).

The study revealed a diagnostic rate of 43.9% for genetic testing. However, some cases that met clinical diagnostic criteria did not receive a conclusive genetic diagnosis. Furthermore, some patients were suspected of having an underlying hereditary condition, although most had inconclusive genetic test results. This could be a result of flaws in the methodology or causes that have not yet been identified. Clinical follow-up is crucial in pediatric liver disease, and further assessments should be conducted when necessary. In addition, genetic testing provides genotype-phenotype correlation and is particularly useful in cases where there are no clear indications or when the patient's condition is challenging to diagnose. However, it is important for clinicians to avoid excessive reliance on genetic testing as it cannot replace routine biochemical and pathology testing in disease management (42).

There are limitations to this study. Firstly, it was a retrospective study conducted in a single center, and no long-term follow-up data on patients were analyzed. Additionally, cases of patients younger than 29 days were excluded from the analysis in our study due to the unavailability of patient data from the neonatal ward. Therefore, these data can only reflect the real-world circumstances of certain patients admitted to the hospital. Secondly, not all children were diagnosed with the same etiology due to the stepwise approach to etiological diagnosis.

In conclusion, our study provides insight into the disease spectrum of pediatric liver disease in a vast population cohort. The fact that over 50% of childhood liver disease originates in infancy, coupled with the low diagnostic rate of liver disease etiology during this period, should be a clinical concern. The causes of liver disease in children are complex and varied. This research validates that different age groups exhibit different etiological profiles. Identifying the etiologies is critical to commence disease-specific management and facilitate the provision of genetic counseling. The more interesting finding is that infection remains the leading cause of pediatric liver disease. Hospital admissions for NAFLD in children have increased rapidly over the past decade, while cytomegalovirus hepatitis has declined markedly.

## Data Availability

The original contributions presented in the study are included in the article/Supplementary Material, further inquiries can be directed to the corresponding author.
